# Why gamers get hooked: the role of reinforcement-based motives and gender in the risk of internet gaming disorder

**DOI:** 10.3389/fpsyg.2026.1843547

**Published:** 2026-09-03

**Authors:** Yuhong Zhou, Zhixiang Yu, Lian Zhou, Xujia Bai, Dan Wu, Xuemei Gao

**Affiliations:** 1 Psychological Research and Counselling Center, Southwest Jiaotong University, Chengdu, China; 2 Faculty of Psychology, Southwest University, Chongqing, China; 3 Xiaguan No.1 Middle School, Dali, China; 4 Changdu No.3 Senior High School, Changdu, China

**Keywords:** gaming motives, gender differences, internet gaming disorder, network comparison, reinforcement perspective

## Abstract

**Introduction:**

Growing attention has been paid to the mechanisms underlying Internet Gaming Disorder (IGD), yet less is known about how reinforcement-based gaming motives are associated with IGD severity and whether these associations differ by gender.

**Methods:**

Guided by opponent process theory and the I-PACE model, this study conducted a cross-sectional questionnaire survey of 1,252 gamers. Network analysis was used to examine the motivational network of IGD, and network comparison analysis was conducted to assess gender differences, with age and game type controlled in the gender-specific analyses.

**Results:**

In the total sample network, IGD severity was most strongly connected with gaming duration. It was also linked to enhancement, social, and coping motive items. Enhancement motives were relatively prominent in the network, while social and coping motives also showed direct associations with IGD severity. Gender comparisons, controlling for age and game type, showed no significant differences in overall network structure or global strength. However, localized gender differences emerged in edges involving social motives, IGD, and gaming duration. Specifically, IGD was more strongly associated with the motive of being sociable in males, whereas it was more closely linked to playing because most friends do in females. The connection between IGD and gaming duration was also stronger in females.

**Discussion:**

These findings suggest that IGD is related not only to gaming time, but also to the psychological needs that gaming may satisfy. They also indicate that gender differences in IGD may be better understood through specific social motive associations rather than broad network level differences.

## 1 Introduction

Internet Gaming Disorder (IGD) is characterized by persistent and uncontrollable gaming behavior that leads to functional impairments, and has been officially recognized as a behavioral addiction (APA, 2013; WHO, 2025). The global prevalence of IGD was non-negligible, estimated at approximately 3.05%, with males being 2.5 times higher than in females (Stevens et al., 2021). Research has found IGD was closely related to some physiological and psychological harms (Evren et al., 2020; Fazeli et al., 2023). Given the absence of substance intake in gaming, individual differences such as gender and motivation may play a critical role in the development of IGD. Although reinforcement mechanisms represent a well-established framework for explaining addiction (Cahalan, 1988), motivational research on IGD from this perspective remains limited, especially in relation to gender differences. Understanding these mechanisms is essential for guiding prevention and intervention efforts.

The opponent process theory of motivation explains the role of reinforcement in addiction through dynamic changes in hedonic, affective, or emotional states (Solomon and Corbit, 1974). The theory proposes two opposing processes. The a-process refers to the initial positive hedonic response to reward. The b-process is a compensatory response that counteracts the a-process and helps restore homeostatic balance. This compensatory process is often associated with negative emotional states. Such progression reflects a shift from reward seeking to relief from negative states, a process well documented in the course of substance addiction (Koob and Moal, 1997; Koob and Volkow, 2010). This shift provides a useful framework for understanding different motives in addiction.

Consistent with this view, empirical studies on substance use and gambling have distinguished among enhancement, coping, and social motives. Enhancement motives reflect pleasure seeking and internal positive reinforcement, coping motives reflect distress relief and internal negative reinforcement, and social motives reflect external positive reinforcement. Only internal motives consistently predict addictive outcomes, with coping showing a direct effect and enhancement acting indirectly via usage (Cooper et al., 1992; Stewart and Zack, 2008; Stewart et al., 2008). As addiction progresses, coping becomes more strongly linked to use, while enhancement remains stable (Cho et al., 2019).

Recent research has extended this view to problematic Internet use. In the latest interpretation of the I-PACE model (Brand et al., 2025), gratification and compensation are both involved in addictive Internet use, but they differ in their relative importance across stages. Gratification is more dominant in recreational use or in the early stage of addictive behaviors, whereas compensation becomes more important in the later stage, as symptoms and negative consequences increase. Gratification refers to positive experiences from the behavior, such as pleasure, reward, or social connection during gaming. Compensation refers to the reduction of negative states, such as anxiety or stress. Thus, gratification corresponds to positive reinforcement, whereas compensation corresponds to negative reinforcement. Wegmann et al. (2025) provided empirical support for this view, showing that risky and pathological Internet users reported higher gratification and compensation than non-problematic users. Notably, pathological users showed the highest compensation, whereas their gratification remained high and did not clearly differ from that of risky users. However, such research in IGD remains limited.

Although few studies have directly examined gaming motives from a reinforcement-based perspective, research on general gaming motives provides relevant evidence. Reviews and meta-analyses have consistently shown that escape motivation has one of the strongest associations with IGD and its symptoms (Bäcklund et al., 2022; Wang and Cheng, 2022). This finding is consistent with the compensation pathway. Supporting this view, Zhou et al. (2023) identified difficulties in regulating negative affect as a proximal factor contributing to IGD. In addition, existing evidence also shows that achievement and social motives are positively associated with IGD, although these associations are weaker than that of escape motivation (Wang and Cheng, 2022). Achievement motives may be linked to gratification, because online games provide reward, competence, progress, and success (Demetrovics et al., 2011). Social motives may also reflect gratification experience, because online games provide interpersonal reward, peer interaction, online social capital, and a sense of belonging (Demetrovics et al., 2011; Heng et al., 2021). Taken together, these findings suggest that compensation-related motives may be particularly salient in IGD, while gratification-related motives also contribute to IGD risk.

However, existing gaming-specific motivation instruments assess a broad range of motives, including achievement, immersion, social interaction, escape, coping, and recreation (Yee, 2006; Demetrovics et al., 2011; Wu et al., 2017). Although some of these motives overlap with reinforcement processes, to our knowledge, no gaming-specific measure has been developed directly around internal positive reinforcement, external positive reinforcement, and internal negative reinforcement, or the related gratification–compensation distinction. A measure organized around these reinforcement functions could complement broader gaming-motivation instruments by enabling a more direct examination of how gratification- and compensation-related motives are associated with IGD severity. Such research may enhance our understanding of how distinct reinforcement mechanisms correlate with IGD, offering a more nuanced framework for future clinical and empirical research.

Gender differences are also important for understanding the motivational structure of IGD. Although males generally show a higher risk of IGD, gender differences may not be limited to prevalence or symptom severity (Stevens et al., 2021; Dong and Potenza, 2022). Recent IGD research suggests that males may be more sensitive to gaming-related rewards and show stronger cue-induced craving during gaming. In contrast, females with IGD may show stronger links with negative mood, loneliness, emotional dysregulation, and affective problems (Dong and Potenza, 2022; Dong et al., 2018a, b; Wang et al., 2022). In addition, research also finds that males are often more likely to play for competitive or achievement-related reasons, whereas females may be more likely to play for social and relational reasons (Martucci et al., 2023; Gisbert-Pérez et al., 2024). Together, these findings suggest that IGD risk may involve different motivational functions across gender, with male gamers more closely linked to gratification-related motives and female gamers more closely linked to coping and social-relational motives. However, such research has received limited empirical attention to date.

### 1.1 The current study

To address these issues, the present study constructed a reinforcement-based motivational network model of IGD. First, we aimed to identify the gaming motives most directly connected to IGD severity and the most central motives in the network. Second, we examined whether these network characteristics differed by gender. Based on the theoretical framework and prior evidence, two hypotheses were proposed:

H1: Compensation-related motives, especially coping motives, would be strongly connected to IGD severity, while gratification-related motives, including enhancement and social motives, would also be positively connected to IGD severity. In addition, coping-related motives were expected to show relatively high centrality in the IGD motivational network.

H2: The motivational network of IGD would differ by gender. IGD risk among males was expected to be more closely related to gratification-related motives, especially enhancement motives, whereas IGD risk among females was expected to be more closely related to compensation-related and social-relational motives.

## 2 Materials and methods

### 2.1 Participants and procedures

We conducted a cross-sectional survey among gamers using the Wenjuanxing platform in China. Participants provided informed consent at the beginning of the survey. A total of 1,427 individuals participated. Of these, 131 were excluded for failing both attention-check items. Considering the necessity of 8 h of sleep, 26 participants who reported an average daily gaming time exceeding 16 h on either weekday or weekend were excluded. Furthermore, 18 participants were excluded for reporting an average weekly gaming time of less than 2 h. The final sample comprised 1,252 participants (64.30% males), aged 15 to 30 years (*M* = 20.99, *SD* = 2.84). The Institutional Review Board (IRB) approved this study.

### 2.2 Measures

#### 2.2.1 Gaming motives questionnaire- revised

Because no existing gaming-specific motivation measure was explicitly developed within a positive and negative reinforcement framework, we adapted the Gambling Motives Questionnaire (GMQ; Stewart and Zack, 2008) to the gaming context. The resulting Gaming Motives Questionnaire-Revised (GMQ-R) assesses three reinforcement-based motives: coping (internal negative reinforcement; COP), enhancement (internal positive reinforcement; ENH), and social motives (external positive reinforcement; SOC). This framework is consistent with reinforcement-based accounts of addictive behaviors and with recent interpretations of the I-PACE model, which distinguish gratification-related positive reinforcement from compensation-related negative reinforcement (Brand et al., 2025). Given the conceptual parallels between gambling disorder and IGD as behavioral addictions (González-Cabrera et al., 2023), the GMQ provides a theoretically appropriate source instrument for measuring similar motivational dimensions in gaming.

To adapt the GMQ to the gaming context, all references to “gambling” were replaced with “gaming,” while the original item meanings, response format, and theoretical three-factor structure were retained. The psychometric properties of the adapted GMQ-R were evaluated in the present sample. The total sample was randomly divided into an exploratory factor analysis (EFA) sample (*n* = 626) and a confirmatory factor analysis (CFA) sample (*n* = 626). In the EFA sample, the data were suitable for factor analysis, KMO = 0.94, Bartlett’s test of sphericity, χ^2^(105) = 3968.67, *p* < 0.001. Following the structure of the original GMQ, principal component analysis with promax rotation was conducted with the number of components fixed to three. Items were considered for deletion if they showed a maximum loading below 0.40, a communality below 0.30, substantial cross-loading, or a primary loading on a component inconsistent with their original GMQ theoretical dimension. Substantial cross-loading was defined as a secondary loading of 0.40 or higher with a primary-secondary loading difference below 0.10 (Acar Güvendir and Özer Özkan, 2022; Howard, 2016). This iterative procedure led to the removal of GMQ13 because of its low maximum loading and GMQ8 because it loaded primarily on an unintended component. The retained 13 items accounted for 60.52% of the total variance.

In the independent CFA sample, the retained 13-item three-factor model, specified according to the original GMQ three-factor structure of social, coping, and enhancement motives, showed good fit, χ^2^(62) = 145.93, χ^2^/df = 2.35, CFI = 0.97, TLI = 0.96, RMSEA = 0.05, SRM*R* = 0.03. The corresponding one-factor model showed poorer fit than the three-factor model, supporting the theoretically specified multidimensional structure. The final 13-item GMQ-R comprised three subscales: social motives (4 items), coping motives (4 items), and enhancement motives (5 items). Details of the retained items, factor loadings, full EFA and CFA results, as well as the correspondence between the original GMQ item numbers and the renumbered GMQ-R identifiers, are provided in Supplementary Appendix A. In the present study, Cronbach’s *α* values were 0.83, 0.80, and 0.77 for enhancement, social, and coping motives, respectively.

#### 2.2.2 Internet gaming disorder-20 (IGD-20) test

The Internet Gaming Disorder-20 (IGD-20) test (Pontes et al., 2014) was used to assess IGD severity. It includes 20 items rated on a 5-point Likert scale (1 = strongly disagree to 5 = strongly agree), with higher scores indicating greater GD risk. A cutoff score of 71 has been recommended to distinguish disordered from non-disordered gamers. The scale has shown good validity in Chinese samples (Qin et al., 2020), and Cronbach’s alpha in the current study was 0.94.

#### 2.2.3 Demographic and gaming duration

Demographic information, including gender and age, was collected. Gaming duration was assessed with two questions adapted from Zhou et al. (2023). One additional question asked participants to report the type of game they played most frequently.

### 2.3 Data analysis

Before the formal analyses, the psychometric properties of the adapted GMQ-R were evaluated in R 4.1.1 using the readxl, dplyr, tibble, purrr, openxlsx, psych, GPArotation, and lavaan packages. Descriptive statistics, Pearson correlation, and group comparisons were conducted using JASP 0.19.1, with independent-samples t-tests for mean comparisons and two-sample z-tests for proportion differences.

Network analysis and network comparison were conducted in R 4.6.0. The network was estimated using the *bootnet* package with the EBICglasso procedure. Network visualization and centrality estimation were performed using the *qgraph* package. The accuracy of edge weights, edge-weight difference tests, and centrality stability (CS) coefficients were examined using bootstrap procedures implemented in *bootnet*. The CS coefficient reflects the maximum proportion of cases that can be dropped while preserving centrality estimates that remain highly correlated with those of the original network (*r* ≥ 0.70 with 95% certainty). CS Values above 0.50 are considered preferable, whereas values around 0.75 indicate high stability (Epskamp et al., 2018).

For the gender-based network comparison, game type was treated as a six-category nominal variable comprising shooter, role-playing games (RPG), MMORPG, strategy games, MOBA, and other games. Game type was represented by five dummy variables, with shooter games as the reference category. Using complete cases pooled across gender, each of the 15 network nodes, including the 13 GMQ-R items, IGD severity, and gaming duration, was separately regressed on age and the five game-type dummy variables. The resulting unstandardized residuals were then divided by gender and used to estimate the male and female networks based on Pearson correlation matrices. Gender differences in network structure, global strength, individual edge weights, and centrality indices were tested using the *NetworkComparisonTest* package.

For the covariate-adjusted gender-specific network analyses, only participants with complete data on gender, age, game type, and all network nodes were included. Game-type data were missing for 27 participants (19 males and 8 females), and no imputation was performed. These participants were retained in the descriptive analyses and the unadjusted total-sample network but were excluded from the covariate-adjusted gender-specific analyses. Accordingly, the adjusted networks were estimated using data from 786 males and 439 females (*N* = 1,225).

Two centrality indices were reported: strength, which reflects the sum of absolute edge weights, and expected influence, which also considers the sign of edges and is suitable for networks with both positive and negative connections. Betweenness and closeness were excluded due to limited reliability in psychological networks (Bringmann et al., 2019; Epskamp et al., 2018). Edges with an absolute weight of at least 0.03 were considered meaningful for interpretation, following previous network studies (Isvoranu et al., 2021).

## 3 Results

### 3.1 Preliminary analysis

Table 1 presents the descriptive statistics and Pearson’s correlations for the total sample. IGD severity was significantly positively correlated with game duration (*r* = 0.41), enhancement motives (*r* = 0.48), social motives (*r* = 0.44), and coping motives (*r* = 0.42). Game duration was also significantly positively correlated with all three motive dimensions (r_s_ = 0.21–0.28). The three reinforcement-based motives were significantly positively intercorrelated (r_s_ = 0.49–0.73). These results indicate that higher IGD severity was associated with longer gaming duration and stronger reinforcement-based gaming motives.

**Table 1:** Descriptive statistics and Pearson’s correlations in total sample.

Variables	Gender	Age	Gaming duration	IGD	ENH	SOC	COP
Age	0.03						
Gaming duration	0.18^***^	−0.02					
IGD	0.16^***^	0.06^*^	0.41^***^				
ENH	0.14^***^	0.15^***^	0.28^***^	0.48^***^			
SOC	0.24^***^	0.11^***^	0.28^***^	0.44^***^	0.54^***^		
COP	0.11^***^	0.15^***^	0.21^***^	0.42^***^	0.73^***^	0.49^***^	
Mean	-	20.99	21.90	60.34	3.04	2.59	3.00
*SD*	-	2.84	12.48	17.12	0.62	0.75	0.63

ENH, enhancement motives; SOC, social motives; COP, coping motives. Gender was coded as 0 = female and 1 = male. ^*^p < 0.05; ^**^p < 0.01; ^***^p < 0.001.

Gender comparisons further showed that males reported significantly longer game duration, higher IGD severity, and higher scores on enhancement, social, and coping motives than females (Table 2). Gender differences were also observed in game type preferences. Males were more likely to report shooter and MOBA games, whereas females were more likely to report RPGs and games in the “Others” category. These findings support the need to further examine gender-specific motivational networks of IGD.

**Table 2:** Descriptive statistics and group differences by gender across all measures.

Variables	Males (*n* = 805)	Females (*n* = 447)	*t/Z*	Cohen’s d/RD
Age	21.05 ± 2.92	20.88 ± 2.69	1.00	0.059
Game duration	23.56 ± 12.87	18.92 ± 11.17	6.40^***^	0.378
IGD	62.33 ± 16.41	56.77 ± 17.79	5.57^***^	0.329
ENH	3.10 ± 0.58	2.92 ± 0.66	4.94^***^	0.291
SOC	2.72 ± 0.70	2.35 ± 0.76	8.84^***^	0.521
COP	3.05 ± 0.62	2.91 ± 0.66	3.80^***^	0.224
Game type				
SHOOTER	22.73%	15.21%	3.18^*^	0.075
RPGs	3.73%	7.61%	−2.99^*^	−0.038
MMORPG	2.24%	1.57%	0.81	0.007
STRATEGY	1.99%	2.24%	−0.30	−0.003
MOBA	64.35%	55.93%	2.93^*^	0.084
Others	2.61%	15.66%	−8.52^*^	−0.131
Missing	2.36%	1.79%	0.67	0.006

Values for continuous variables are presented as M ± SD, and values for game type are presented as percentages. For continuous variables, independent-samples t-tests were used, with Cohen’s d reported as the effect size. For categorical variables, namely game-type comparisons, two-sample z-tests for proportions were used. RD = risk difference, calculated as the male proportion minus the female proportion; positive RD values indicate higher proportions among males, whereas negative values indicate higher proportions among females. For game-type comparisons, significance was evaluated after Bonferroni correction across the six substantive game categories. Missing was reported for descriptive purposes only. ^*^p < 0.05; ^**^p < 0.01; ^***^p < 0.001.

### 3.2 Motivational network of IGD in total sample

**Figure 1:**
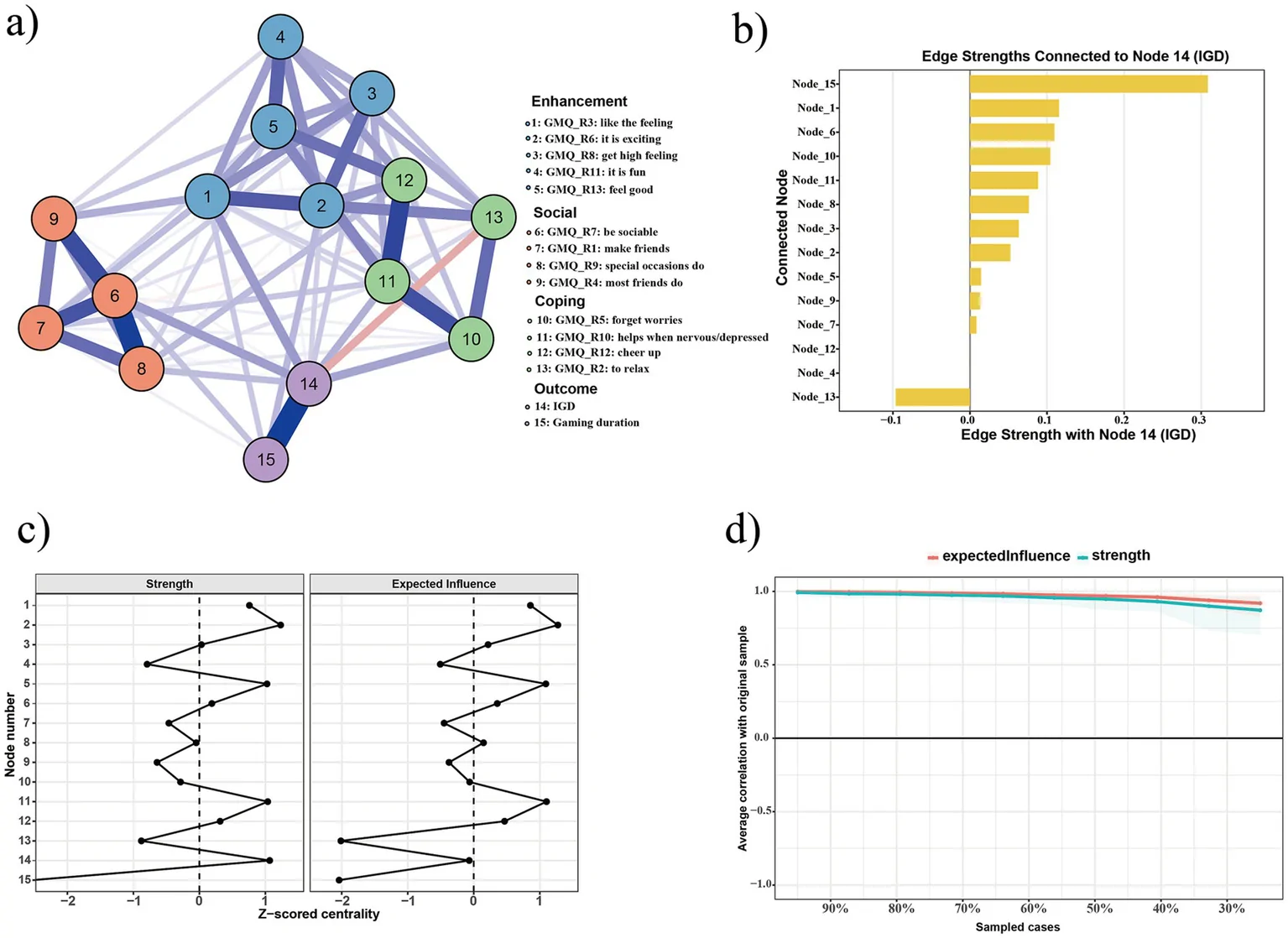
Motivational network of IGD (*N* = 1,252) and related measures. **(a)** shows the motivational network of IGD in total sample. Edges represent regularized partial correlations, with thicker edges indicating stronger associations. Blue edges indicate positive associations, whereas red edges indicate negative associations. Panel **(b)** presents the edge weights connected to Node 14, namely IGD. **(c)** shows the standardized centrality estimates for strength and expected influence. **(d)** presents the case dropping bootstrap results for centrality stability. Nodes 1 to 5 represent enhancement motives: 1 = GMQ_R3: Like the feeling, 2 = GMQ_R6: It is exciting, 3 = GMQ_R8: Get high feeling, 4 = GMQ_R11: It is fun, and 5 = GMQ_R13: Feel good. Nodes 6 to 9 represent social motives: 6 = GMQ_R7: Be sociable, 7 = GMQ_R1: Make friends, 8 = GMQ_R9: Special occasions, and 9 = GMQ_R4: Most friends do. Nodes 10 to 13 represent coping motives: 10 = GMQ_R5: Forget worries, 11 = GMQ_R10: Helps when nervous or depressed, 12 = GMQ_R12: Cheer up, and 13 = GMQ_R2: To relax. Node 14 represents IGD, and Node 15 represents gaming duration.

As shown in Figure 1, the network (Figure 1a) revealed positive partial correlations among ENH, SOC and COP and their associations with IGD, except for node 13 (GMQ-R2: “to relax”), which showed a weak negative link (*r* = −0.10). Among all connections with IGD (Figure 1b), gaming duration showed the strongest connection (*r* = 0.31), followed by GMQ-R3 (ENH: “like the feeling,” *r* = 0.12), GMQ-R7 (SOC: “be sociable,” *r* = 0.11), and GMQ-R5 (COP: “forget worries,” *r* = 0.10). In addition, weaker connections with IGD were observed for other nodes within each dimension: GMQ-R10 (COP: “helps w`hen nervous/depressed” *r* = 0.09), GMQ-R9 (SOC: “special occasions do” *r* = 0.08), GMQ-R8 (ENH: “get high feeling” *r* = 0.06), and GMQ-R6 (ENH: “it is exciting,” *r* = 0.05).

The centrality results showed that GMQ-R6 (“it is exciting,” ENH) had the highest strength centrality (1.07) and expected influence (1.07) in the total-sample network (Figure 1c), indicating that this item had the largest overall sum of direct associations with other nodes in the network. The CS coefficients (Figure 1d) indicated good overall network stability for both strength and expected influence (strength = 0.75, EI = 0.75). These findings suggest that IGD severity in the total sample was associated with internal positive reinforcement, especially enhancement related motives. These findings partially support H1.

### 3.3 Comparison of motivational network of IGD between male and female samples

**Figure 2:**
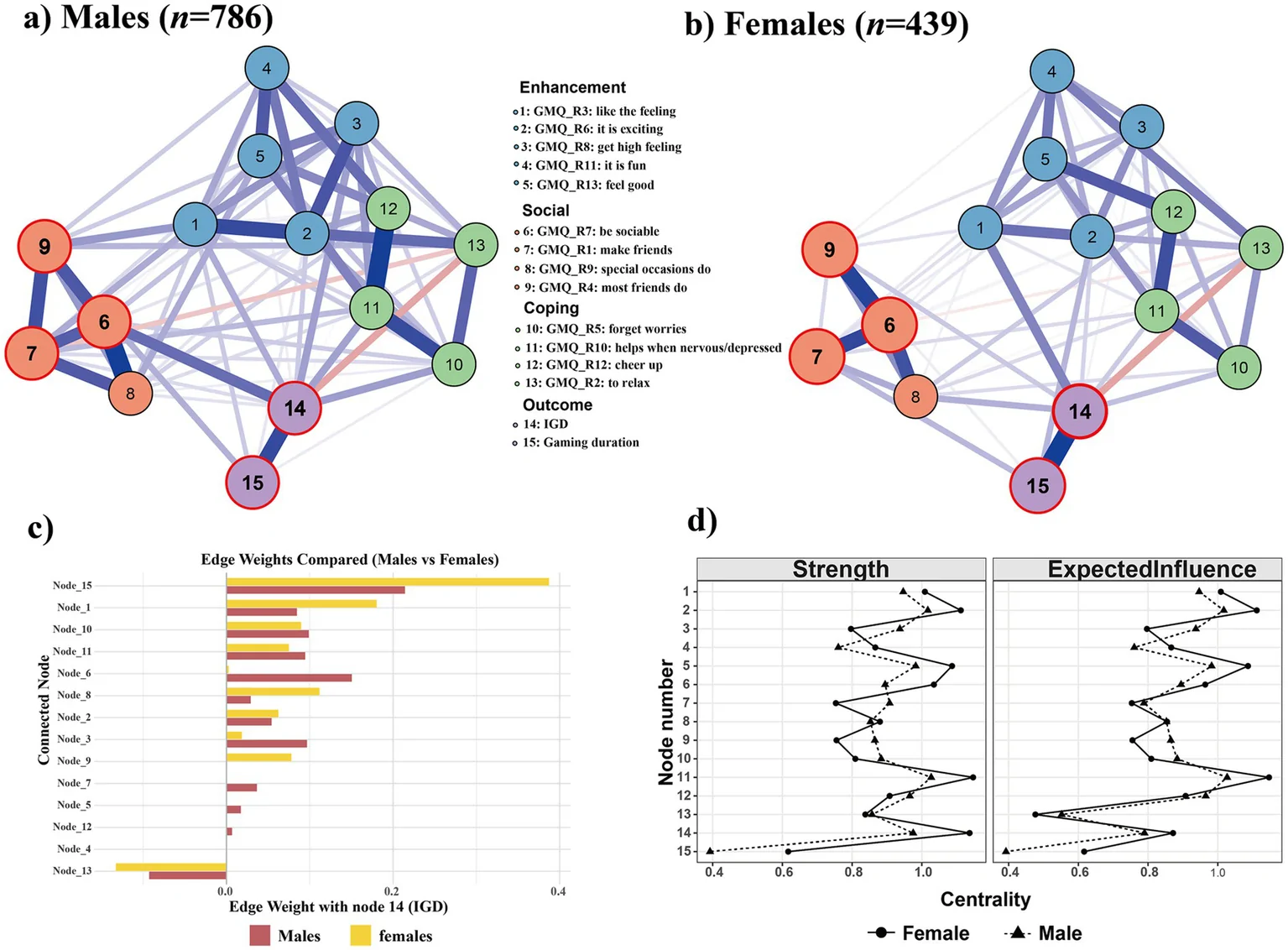
Motivational network of IGD across gender. **(a,b)** show the covariate-adjusted motivational networks for male (*n* = 786) and female (*n* = 439) players, respectively. All network nodes were residualized for age and game type before network estimation. Node colors indicate motivational domains and outcome variables. Edges represent regularized partial correlations, with thicker edges indicating stronger associations. Blue edges indicate positive associations, whereas red edges indicate negative associations. For visualization purposes, IGD, gaming duration, and nodes involved in edges showing gender differences connected to IGD or gaming duration were manually enlarged and outlined in red. **(c)** presents the edge weights connected to IGD across male and female networks. **(d)** shows strength and expected influence for each node.

After excluding 27 participants with missing game-type data, the covariate-adjusted gender-specific analyses included 786 males and 439 females. After controlling for age and game type, comparison of the motivational networks across gender (Figure 2) revealed no significant difference in overall network structure between males and females, *p* = 0.09. The global strength also did not differ significantly between the two networks (male = 6.63, female = 6.87), *p* = 0.30.

At the edge level, four edges involving either IGD or gaming duration differed between males and females. The edge between GMQ-R7 (“be sociable,” SOC) and IGD was stronger in males (*r* = 0.15) than in females (*r* = 0.003), *p* < 0.01. In contrast, three edges were stronger in females than in males. Specifically, the edge between GMQ-R4 (“most friends do,” SOC) and IGD was present in the female network (*r* = 0.08) but absent in the male network, *p* < 0.05. The connection between IGD and gaming duration was stronger in females (*r* = 0.39) than in males (*r* = 0.21), *p* < 0.01. In addition, the edge between GMQ-R1 (“make friends,” SOC) and gaming duration was present in the female network (*r* = 0.10) but absent in the male network, *p* < 0.05.

Overall, gender differences were mainly reflected in social-motive-related pathways. In males, IGD was more strongly associated with the motive of being sociable, whereas in females, IGD was more closely linked to playing because most friends do, suggesting a stronger role of maintaining peer relationships. In addition, the stronger connection between IGD and gaming duration in females further suggests that gaming time may be more closely involved in IGD processes among female players rather than males. Thus, H2 was not supported.

## 4 Discussion

This study constructed a reinforcement-based motivational network of IGD and examined whether this network differed by gender. In the total sample, IGD severity was directly connected with gaming duration and several motive items reflecting enhancement, social, and coping functions. Gender comparisons showed no significant differences in overall network structure or global strength. However, among the focal edges connecting IGD or gaming duration with gaming motive items, gender differences were observed only for social motive items. Together, these findings suggest that IGD should not be understood solely as excessive gaming behavior, but also in relation to the psychological needs that gaming may satisfy, such as enjoyment, social connection, or relief from negative mood. Moreover, gender differences in IGD may be reflected more in specific motivational associations, especially those involving social motives, than in broad network level differences.

The findings from the total sample provide partial support for H1. Gaming duration showed the strongest direct connection with IGD severity, indicating that behavioral involvement remains closely associated with IGD symptoms. However, gaming duration had the lowest centrality in the network, suggesting that it was less embedded in the broader motivational structure. In contrast, the enhancement item “like the feeling” showed the strongest motive related connection with IGD after gaming duration, and another enhancement item, “it is exciting,” showed the highest strength and expected influence. This pattern suggests that enhancement related motives were not only associated with IGD severity, but also more broadly connected with other motives in the network. Similar findings were reported by Zhou et al. (2023), who found that game duration was strongly linked to IGD but showed relatively weak centrality, whereas motivational factors appeared to be more proximal correlates of IGD.

However, IGD severity was still connected with items from all three reinforcement-based motive domains, suggesting that enhancement, social, and coping motives may all be relevant to IGD. This pattern is broadly consistent with the I-PACE framework, which suggests that both gratification and compensation may be involved in addictive Internet use (Brand et al., 2025; Wegmann et al., 2025). One possible explanation for the relatively central position of enhancement related motives is that the present nonclinical sample may include many risky rather than clinically addicted gamers. In such samples, gratification related experiences, such as enjoyment and excitement, may remain salient, whereas compensation related processes may become more prominent as symptoms and negative consequences increase.

The gender comparison did not support H2 as originally expected. We expected that IGD among males would be more closely related to gratification related motives, especially enhancement motives, whereas IGD among females would be more closely related to compensation related motives and social motives. However, the observed gender differences were mainly found in the focal edges involving IGD or gaming duration, and these differences were largely related to social motive items. Specifically, IGD was more strongly associated with the motive of being sociable in males, whereas in females, IGD was more closely linked to playing because most friends do. In addition, the connection between IGD and gaming duration was stronger in females, and the motive of making friends was connected with gaming duration only in the female network.

These findings suggest that social motives may be relevant to IGD in both male and female players, but the specific social contexts may differ. For male players, the stronger link between being sociable and IGD may indicate that gaming is associated with opportunities for social engagement or social expansion, which is consistent with evidence that social motivation and sociability are associated with online game addiction (Blinka and Mikuška, 2014). This interpretation is also consistent with research showing that in game social interaction and online social capital are related to gaming disorder (Heng et al., 2021). For female players, the stronger link between playing because most friends do and IGD may indicate that gaming is more embedded in existing peer contexts. This interpretation is consistent with prior work suggesting that female players may be more likely to engage in gaming for social and relational purposes (Gisbert-Pérez et al., 2024; Martucci et al., 2023). The stronger association between IGD and gaming duration in females further suggests that gaming time may be more closely tied to IGD severity among female players. More broadly, these findings align with recent arguments that gender differences in IGD may involve not only symptom severity or prevalence, but also different motivational and psychosocial contexts of gaming (Dong and Potenza, 2022).

## 5 Limitations and implications

This study has several limitations. First, the cross-sectional design precludes causal conclusions. The network edges represent conditional associations among variables and cannot determine the temporal direction between gaming motives, gaming duration, and IGD severity. Future longitudinal studies are needed to examine whether reinforcement-based gaming motives change across different stages of IGD. Second, the telescoping effect (i.e., females begin addictive behaviors later than males but progress more quickly) has been found in various types of substance addiction (Fonseca et al., 2021). Whether this pattern also applies to IGD need to be explored in future research. Third, the sample consisted of Chinese gamers, and MOBA games represented the most common game type in both gender groups. Therefore, the generalizability of the findings to other cultural contexts, age groups, and game genres should be examined in future research.

Despite these limitations, the present study has several implications. First, the findings extend reinforcement-based accounts of IGD by showing that enhancement, social, and coping motives were associated with IGD severity. Second, the adapted GMQ_R scale showed acceptable psychometric properties, suggesting that the reinforcement-based motive structure may be applicable to the study of gaming behavior, although further validation is still needed. Third, the findings may inform prevention and intervention by encouraging assessment of not only gaming duration, but also the psychological needs that gaming may satisfy, such as enjoyment, social connection, or relief from negative mood. Fourth, the gender findings suggest that intervention efforts should consider specific motive patterns across gender rather than rely on broad gender stereotypes.

## 6 Conclusion

This study examined how reinforcement-based gaming motives were associated with IGD severity and whether these associations differed by gender. In the total sample, IGD severity was linked to both gaming duration and reinforcement-based motives, especially enhancement motives. Social and coping motives also showed direct associations with IGD severity. Gender differences were not found in overall network structure or global strength. Instead, localized differences emerged in edges involving social motives, IGD, and gaming duration. These findings suggest that IGD is related not only to gaming time, but also to the psychological needs that gaming may satisfy. They also suggest that gender differences in IGD may be better understood through specific social motive associations rather than broad network differences.

## Data Availability

The original contributions presented in the study are included in the article/Supplementary material. Further inquiries can be directed to the corresponding author.

## References

[ref9001] Acar Güvendir M. Özer Özkan Y. (2022). Item Removal Strategies Conducted in Exploratory Factor Analysis: A Comparative Study. Int. J. Assess. Tool. Educ. 9, 165–180. doi: 10.21449/ijate.827950

[ref1] APA (2013). In Diagnostic and Statistical Manual of mental Disorders: DSM-5. 5th Edn Washington, DC: American Psychiatric Association.

[ref2] Bäcklund C. Elbe P. Gavelin H. M. Sörman D. E. Ljungberg J. K. (2022). Gaming motivations and gaming disorder symptoms: a systematic review and meta-analysis. J. Behav. Addict. 11, 667–688. doi: 10.1556/2006.2022.00053, 36094861 PMC9872536

[ref6] Blinka L. Mikuška J. (2014). The role of social motivation and sociability of gamers in online game addiction. Journal of Psychosocial Research on Cyberspace 8:6. doi: 10.5817/cp2014-2-6

[ref7] Brand M. Müller A. Wegmann E. Antons S. Brandtner A. Müller S. M. . (2025). Current interpretations of the I-PACE model of behavioral addictions. J. Behav. Addict. 14, 1–17. doi: 10.1556/2006.2025.00020, 40063161 PMC11974429

[ref9] Bringmann L. F. Elmer T. Epskamp S. Krause R. W. Schoch D. Wichers M. . (2019). What do centrality measures measure in psychological networks? J. Abnorm. Psychol. 128, 892–903. doi: 10.1037/abn0000446 31318245

[ref11] Cahalan D. (1988). Implications of the disease concept of alcoholism. Drugs Soc. 2, 49–68. doi: 10.1300/J023v02n03_04

[ref14] Cho S. B. Su J. Kuo S. I. Bucholz K. K. Chan G. Edenberg H. J. . (2019). Positive and negative reinforcement are differentially associated with alcohol consumption as a function of alcohol dependence. Psychol. Addict. Behav. 33:58. doi: 10.1037/adb0000436 30667237 PMC6459181

[ref16] Cooper M. L. Russell M. Skinner J. B. Windle M. (1992). Development and validation of a three-dimensional measure of drinking motives. Psychol. Assess. 4, 123–132. doi: 10.1037/1040-3590.4.2.123

[ref17] Demetrovics Z. Urbán R. Nagygyörgy K. Farkas J. Zilahy D. Mervó B. . (2011). Why do you play? The development of the motives for online gaming questionnaire (MOGQ). Behav. Res. Methods 43, 814–825. doi: 10.3758/s13428-011-0091-y 21487899

[ref18] Dong G.-H. Potenza M. N. (2022). Considering gender differences in the study and treatment of internet gaming disorder. J. Psychiatr. Res. 153, 25–29. doi: 10.1016/j.jpsychires.2022.06.057, 35793576

[ref19] Dong G. Wang L. Du X. Potenza M. N. (2018a). Gender-related differences in neural responses to gaming cues before and after gaming: implications for gender-specific vulnerabilities to internet gaming disorder. Soc. Cogn. Affect. Neurosci. 13, 1203–1214. doi: 10.1093/scan/nsy084, 30272247 PMC6234325

[ref21] Dong G. Zheng H. Liu X. Wang Y. Du X. Potenza M. N. (2018b). Gender-related differences in cue-elicited cravings in internet gaming disorder: the effects of deprivation. J. Behav. Addict. 7, 953–964. doi: 10.1556/2006.7.2018.118, 30556781 PMC6376376

[ref23] Epskamp S. Borsboom D. Fried E. I. (2018). Estimating psychological networks and their accuracy: a tutorial paper. Behav. Res. Methods 50, 195–212. doi: 10.3758/s13428-017-0862-1, 28342071 PMC5809547

[ref24] Evren C. Evren B. Dalbudak E. Topcu M. Kutlu N. (2020). Relationship of internet gaming disorder symptom severity with non-suicidal self-injury among young adults. Dusunen Adam 33, 79–86. doi: 10.14744/DAJPNS.2019.00063, 30896977

[ref25] Fazeli S. Zeidi I. M. Lin C.-Y. Namdar P. Griffiths M. D. Ahorsu D. K. . (2023). Depression, anxiety, and stress mediate the associations between internet gaming disorder, insomnia, and quality of life during the COVID-19 outbreak. Addict. Behav. Rep. 12:100307. doi: 10.1016/j.abrep.2020.100307 PMC7581367 33110934

[ref27] Fonseca F. Robles-Martínez M. Tirado-Muñoz J. Alías-Ferri M. Mestre-Pintó J.-I. Coratu A. M. . (2021). A gender perspective of addictive disorders. Curr. Addict. Rep. 8, 89–99. doi: 10.1007/s40429-021-00357-9, 33614395 PMC7885978

[ref28] Gisbert-Pérez J. Martí-Vilar M. Merino-Soto C. Chans G. M. Badenes-Ribera L. (2024). Gender differences in internet gaming among university students: a discriminant analysis. Front. Psychol. 15:1412739. doi: 10.3389/fpsyg.2024.1412739, 39569103 PMC11577638

[ref29] González-Cabrera J. Basterra-González A. Ortega-Barón J. Caba-Machado V. Díaz-López A. Pontes H. M. . (2023). Loot box purchases and their relationship with internet gaming disorder and online gambling disorder in adolescents: a prospective study. Comput. Hum. Behav. 143:107685. doi: 10.1016/j.chb.2023.107685

[ref32] Heng S. Zhao H. Wang M. (2021). In-game social interaction and gaming disorder: a perspective from online social capital. Front. Psych. 11:468115. doi: 10.3389/fpsyt.2020.468115, 33613329 PMC7886677

[ref9003] Howard M. C. (2016). A Review of Exploratory Factor Analysis Decisions and Overview of Current Practices: What We Are Doing and How Can We Improve? International Journal of Human-Computer Interaction, 32, 51–62. doi: 10.1080/10447318.2015.1087664

[ref33] Isvoranu A.-M. Boyette L.-L. Guloksuz S. Borsboom D. (2021). “Symptom network models of psychosis,” in Psychotic Disorders: Comprehensive Conceptualization and Treatments, eds C. A. Tamminga, E. I. Ivleva, U. Reininghaus, and J. van Os (Oxford University Press), 70–78.

[ref34] Koob G. F. Moal M. L. (1997). Drug abuse: hedonic homeostatic dysregulation. Science 278, 52–58. doi: 10.1126/science.278.5335.52, 9311926

[ref35] Koob G. F. Volkow N. D. (2010). Neurocircuitry of addiction. Neuropsychopharmacology 35, 217–238. doi: 10.1038/npp.2009.110, 19710631 PMC2805560

[ref38] Martucci A. Gursesli M. C. Duradoni M. Guazzini A. (2023). Overviewing gaming motivation and its associated psychological and sociodemographic variables: a PRISMA systematic review. Hum. Behav. Emerg. Technol. 2023, 1–156. doi: 10.1155/2023/5640258

[ref9004] Pontes H. M. Király O. Demetrovics Z. Griffiths M. D. (2014). The conceptualisation and measurement of DSM-5 internet gaming disorder: The development of the IGD-20 test. PLoS ONE, 9, e110137. doi: 10.1371/journal.pone.0110137 25313515 PMC4196957

[ref9002] Qin L. Liu Q. Luo T. (2020). Reliability and validity of the 20-item Internet Gaming Disorder Test for Chinese college students. Chin. J. Clin. Psychol. 28, 33–36. doi: 10.16128/j.cnki.1005-3611.2020.01.008

[ref41] Solomon R. L. Corbit J. D. (1974). An opponent-process theory of motivation: I Temporal dynamics of affect. Psychological Review 81, 119–145. doi: 10.1037/h0036128, 4817611

[ref42] Stevens M. W. Dorstyn D. Delfabbro P. H. King D. L. (2021). Global prevalence of gaming disorder: a systematic review and meta-analysis. Aust. N. Z. J. Psychiatry 55, 553–568. doi: 10.1177/0004867420962851, 33028074

[ref43] Stewart S. H. Zack M. (2008). Development and psychometric evaluation of a three-dimensional gambling motives questionnaire. Addiction 103, 1110–1117. doi: 10.1111/j.1360-0443.2008.02235.x, 18554344

[ref44] Stewart S. H. Zack M. Collins P. Klein R. M. Fragopoulos F. (2008). Subtyping pathological gamblers on the basis of affective motivations for gambling: relations to gambling problems, drinking problems, and affective motivations for drinking. Psychol. Addict. Behav. 22, 257–268. doi: 10.1037/0893-164X.22.2.257, 18540723

[ref48] Wang H.-Y. Cheng C. (2022). The associations between gaming motivation and internet gaming disorder: systematic review and meta-analysis. JMIR Mental Health 9:e23700. doi: 10.2196/23700, 35175204 PMC8895288

[ref49] Wang Z.-L. Song K.-R. Zhou N. Potenza M. N. Zhang J.-T. Dong G.-H. (2022). Gender-related differences in involvement of addiction brain networks in internet gaming disorder: relationships with craving and emotional regulation. Prog. Neuro-Psychopharmacol. Biol. Psychiatry 118:110574. doi: 10.1016/j.pnpbp.2022.110574, 35569619

[ref50] Wegmann E. Antons S. Schmidt L. D. Klein L. Montag C. Rumpf H. J. . (2025). Feels good, and less bad: problematic use of the internet is associated with heightened experiences of both gratification and compensation [article early access]. J. Behav. Addict. 14, 263–275. doi: 10.1556/2006.2024.00067, 39786377 PMC11974442

[ref52] WHO (2025). ICD-11 for Mortality and Morbidity Statistics, 2025-01 Release: Gaming Disorder. Geneva: World Health Organization. Available online at: https://icd.who.int/browse/2025-01/mms/en#1448597234

[ref53] Wu A. M. S. Lai M. H. C. Yu S. Lau J. T. F. Lei M. (2017). Motives for online gaming questionnaire: its psychometric properties and correlation with internet gaming disorder symptoms among Chinese people. J. Behav. Addict. 6, 11–20. doi: 10.1556/2006.6.2017.007, 28264590 PMC5572999

[ref54] Yee N. (2006). Motivations for play in online games. Cyberpsychol. Behav. 9, 772–775. doi: 10.1089/cpb.2006.9.772, 17201605

[ref56] Zhou Y. Lv X. Wang L. Li J. Gao X. (2023). What increases the risk of gamers being addicted? An integrated network model of personality–emotion–motivation of gaming disorder. Comput. Hum. Behav. 141:107647. doi: 10.1016/j.chb.2022.107647

