# Supplementary material for: Why gamers get hooked: the role of reinforcement-based motives and gender in the risk of internet gaming disorder
Source: Front Psychol. 2026 Sep 3;17:1843547. doi: 10.3389/fpsyg.2026.1843547 (PMC13582440; doi:10.3389/fpsyg.2026.1843547)
Supplement: Supplementary file 1 [file Supplementary_file_1.docx]

**Appendix A. Psychometric evaluation of the adapted GMQ-R**

The total sample (*N* = 1252) was randomly divided into two equal subsamples, with Sample 1 used for EFA and Sample 2 used for CFA. Sample 1 included 626 participants, of whom 405 were male and 221 were female; their ages ranged from 15 to 30 years (*M* = 21.04, *SD* = 2.87). As shown in Tables A1–A3, the iterative EFA supported a three-factor structure broadly corresponding to the original GMQ dimensions of enhancement, social, and coping motives. In the initial 15-item solution, most items loaded primarily on their intended components; however, GMQ13 showed a maximum loading below 0.40 and was therefore removed. After removing GMQ13, the 14-item solution still showed a generally clear three-factor structure, but GMQ8, originally assigned to the coping subscale, loaded primarily on the social component rather than the coping component, indicating a mismatch between its empirical loading pattern and its original theoretical assignment. GMQ8 was therefore removed. In the final 13-item solution, all retained items loaded above 0.40 on their intended components, with enhancement items loading on Factor 1, social items loading on Factor 2, and coping items loading on Factor 3. The final solution showed clear primary loadings for enhancement motives (0.477–0.854), social motives (0.686–0.816), and coping motives (0.565–0.921), with communalities ranging from 0.499 to 0.695.

**Table A1**. EFA: Factor loadings of the 15 original GMQ items.

|  | Factor 1:  Enhancement motives | Factor 2:  Social motives | Factor 3:  Coping motives | Communalities |
| --- | --- | --- | --- | --- |
| **Enhancement motives subscale** |  |  |  |  |
| 3. Because you like the feeling | **0.870** | 0.122 | -0.143 | 0.664 |
| 12. Because it’s fun | **0.843** | -0.111 | -0.034 | 0.582 |
| 6. Because it’s exciting | **0.703** | -0.040 | 0.122 | 0.595 |
| 9. To get a “high” feeling | **0.697** | 0.074 | 0.014 | 0.564 |
| 15. Because it makes you feel good | **0.449** | 0.102 | 0.310 | 0.590 |
| **Social motives subscale** |  |  |  |  |
| 7. To be sociable | -0.079 | **0.847** | -0.015 | 0.636 |
| 1. As a way to make friends | 0.135 | **0.765** | -0.143 | 0.597 |
| 10. Because it’s something I do on special occasions | -0.206 | **0.747** | 0.242 | 0.608 |
| 4. Because it’s what most of your friends do when you get together | 0.111 | **0.685** | -0.056 | 0.518 |
| 13. Because it makes a social gathering more enjoyable | 0.161 | 0.356 | 0.307 | 0.498 |
| **Coping motives subscale** |  |  |  |  |
| 5. To forget your worries | -0.207 | 0.054 | **0.916** | 0.658 |
| 11. Because it helps when you are feeling nervous or depressed | 0.018 | 0.093 | **0.746** | 0.660 |
| 14. To cheer up when you’re in a bad mood | 0.036 | 0.082 | **0.696** | 0.593 |
| 2. To relax | 0.305 | -0.338 | **0.613** | 0.508 |
| 8. Because you feel more self-confident or sure of yourself | 0.248 | **0.554** | 0.068 | 0.592 |

**Note**. Bold values indicate factor loadings above 0.40.

**Table A2**. EFA: Factor loadings for the 14-item solution after removing GMQ13

|  | Factor 1:  Enhancement motives | Factor 2:  Social motives | Factor 3:  Coping motives | Communalities |
| --- | --- | --- | --- | --- |
| **Enhancement motives subscale** |  |  |  |  |
| 3. Because you like the feeling | **0.857** | 0.122 | -0.178 | 0.664 |
| 12. Because it’s fun | **0.845** | -0.106 | -0.042 | 0.587 |
| 9. To get a “high” feeling | **0.693** | 0.072 | 0.020 | 0.560 |
| 6. Because it’s exciting | **0.686** | -0.027 | 0.136 | 0.594 |
| 15. Because it makes you feel good | **0.468** | 0.109 | 0.294 | 0.591 |
| **Social motives subscale** |  |  |  |  |
| 7. To be sociable | -0.082 | **0.837** | 0.023 | 0.650 |
| 10. Because it’s something I do on special occasions | -0.194 | **0.742** | 0.260 | 0.618 |
| 1. As a way to make friends | 0.172 | **0.736** | -0.148 | 0.589 |
| 4. Because it’s what most of your friends do when you get together | 0.130 | **0.670** | -0.050 | 0.522 |
| **Coping motives subscale** |  |  |  |  |
| 5. To forget your worries | -0.201 | 0.084 | **0.908** | 0.679 |
| 11. Because it helps when you are feeling nervous or depressed | 0.047 | 0.110 | **0.717** | 0.657 |
| 14. To cheer up when you’re in a bad mood | 0.071 | 0.098 | **0.663** | 0.589 |
| 2. To relax | 0.310 | -0.314 | **0.591** | 0.509 |
| 8. Because you feel more self-confident or sure of yourself | 0.237 | **0.556** | 0.101 | 0.604 |

**Note**. Bold values indicate factor loadings above 0.40.

**Table A3**. EFA: Final factor loadings for the retained 13-item GMQ-R

|  | Factor 1:  Enhancement motives | Factor 2:  Social motives | Factor 3:  Coping motives | Communalities |
| --- | --- | --- | --- | --- |
| **Enhancement motives subscale** |  |  |  |  |
| 3. Because you like the feeling **(****GMQ_R3)** | **0.854** | 0.132 | -0.175 | 0.666 |
| 12. Because it’s fun **(GMQ_R11)** | **0.843** | -0.083 | -0.057 | 0.591 |
| 9. To get a “high” feeling **(GMQ_R8)** | **0.684** | 0.053 | 0.042 | 0.550 |
| 6. Because it’s exciting **(GMQ_R6)** | **0.682** | -0.022 | 0.138 | 0.594 |
| 15. Because it makes you feel good **(GMQ_R13)** | **0.477** | 0.108 | 0.292 | 0.592 |
| **Social motives subscale** |  |  |  |  |
| 7. To be sociable **(GMQ_R7)** | -0.057 | **0.816** | 0.050 | 0.695 |
| 1. As a way to make friends **(GMQ_R1)** | 0.195 | **0.734** | -0.134 | 0.613 |
| 10. Because it’s something I do on special occasions **(GMQ_R9)** | -0.167 | **0.706** | 0.288 | 0.611 |
| 4. Because it’s what most of your friends do when you get together **(GMQ_R4)** | 0.160 | **0.686** | -0.056 | 0.561 |
| **Coping motives subscale** |  |  |  |  |
| 5. To forget your worries **(GMQ_R5)** | -0.190 | 0.053 | **0.921** | 0.684 |
| 11. Because it helps when you are feeling nervous or depressed **(GMQ_R10)** | 0.062 | 0.097 | **0.718** | 0.657 |
| 14. To cheer up when you’re in a bad mood **(GMQ_R12)** | 0.090 | 0.107 | **0.647** | 0.589 |
| 2. To relax **(GMQ_R2)** | 0.311 | -0.293 | **0.565** | 0.499 |

**Note**. Bold values indicate factor loadings above 0.40.

The CFA was conducted using Sample 2, which included 626 participants, of whom 400 were male and 226 were female; their ages ranged from 15 to 30 years (*M* = 20.93, *SD* = 2.81). The CFA results further supported the retained 13-item three-factor structure of the adapted GMQ-R (see Tables A.4-5). The original 15-item three-factor model showed acceptable but less optimal fit, whereas the EFA-retained 13-item three-factor model showed clearly improved fit, χ²/df = 2.357, CFI = 0.971, TLI = 0.964, RMSEA = 0.047, and SRMR = 0.034. The retained three-factor model also fit substantially better than the corresponding one-factor model. Standardized factor loadings for the retained 13 items were all significant and acceptable, ranging from 0.653 to 0.745 for enhancement motives, from 0.671 to 0.772 for social motives, and from 0.508 to 0.773 for coping motives.

| Model | *χ²/df* | *p* | CFI | TLI | RMSEA | SRMR | AIC | BIC |
| --- | --- | --- | --- | --- | --- | --- | --- | --- |
| Original 15-item one-factor model | 7.393 | <0.001 | 0.839 | 0.812 | 0.101 | 0.072 | 20244.263 | 20444.034 |
| Original 15-item three-factor model | 3.832 | <0.001 | 0.931 | 0.917 | 0.067 | 0.055 | 19874.658 | 20087.747 |
| EFA-retained one-factor model | 8.488 | <0.001 | 0.834 | 0.801 | 0.109 | 0.075 | 17565.805 | 17738.940 |
| EFA-retained three-factor model | 2.357 | <0.001 | 0.971 | 0.964 | 0.047 | 0.034 | 17123.235 | 17309.688 |

**Table A4.** CFA model fit comparison

**Table A5.** Standardized CFA factor loadings for the retained 13-item GMQ-R

| Factor | Item | Standardized loading | *p* |
| --- | --- | --- | --- |
| Enhancement motives | **GMQ_R3** ‘Because you like the feeling’ | 0.680 | <0.001 |
| Enhancement motives | **GMQ_R6** ‘Because it’s exciting’ | 0.745 | <0.001 |
| Enhancement motives | **GMQ_R8** ‘To get a “high” feeling’ | 0.716 | <0.001 |
| Enhancement motives | **GMQ_R11** ‘Because it’s fun’ | 0.653 | <0.001 |
| Enhancement motives | **GMQ_R13** ‘Because it makes you feel good’ | 0.740 | <0.001 |
| Social motives | **GMQ_R1** ‘As a way to make friends’ | 0.691 | <0.001 |
| Social motives | **GMQ_R4** ‘Because it’s what most of your friends do when you get together’ | 0.671 | <0.001 |
| Social motives | **GMQ_R7** ‘To be sociable’ | 0.772 | <0.001 |
| Social motives | **GMQ_R9** ‘Because it’s something I do on special occasions’ | 0.759 | <0.001 |
| Coping motives | **GMQ_R2** ‘To relax’ | 0.508 | <0.001 |
| Coping motives | **GMQ_R5** ‘To forget your worries’ | 0.670 | <0.001 |
| Coping motives | **GMQ_R10** ‘Because it helps when you are feeling nervous or depressed’ | 0.773 | <0.001 |
| Coping motives | **GMQ_R12** ‘To cheer up when you’re in a bad mood’ | 0.773 | <0.001 |

Reliability and criterion-related validity were examined using the full sample, whereas CR and AVE were calculated from the standardized CFA loadings in Sample 2. As summarized in Tables A6 and A7, the retained 13-item GMQ-R also showed adequate reliability and criterion-related validity. Internal consistency was acceptable for enhancement, social, and coping motives, with Cronbach’s α values of 0.830, 0.798, and 0.772, respectively, and McDonald’s ω values of 0.831, 0.799, and 0.775, respectively. Composite reliability was also acceptable across the three subscales, although the AVE for coping motives was slightly below 0.50. Criterion-related validity was supported by significant positive correlations between all three motive dimensions and IGD symptoms as well as gaming duration.

**Table A6.** Reliability, composite reliability, and AVE of the retained 13-item GMQ-R

| Dimensions | Cronbach’s α | McDonald’s ω | CR | AVE |
| --- | --- | --- | --- | --- |
| Enhancement motives | 0.830 | 0.831 | 0.833 | 0.501 |
| Social motives | 0.798 | 0.799 | 0.815 | 0.525 |
| Coping motives | 0.772 | 0.775 | 0.780 | 0.476 |

*Note.* CR = composite reliability; AVE = average variance extracted.

**Table A7**. Criterion-related validity correlations with IGD and gaming duration

|  | IGD | Gaming duration |
| --- | --- | --- |
| Enhancement motives | 0.480^***^ | 0.280^***^ |
| Social motives | 0.444^***^ | 0.280^***^ |
| Coping motives | 0.415^***^ | 0.214^***^ |

*Note.* Values are Pearson correlation coefficients. ^***^ *p* < 0.001.

**Table A8**. Correspondence between the original GMQ item numbers and the final GMQ-R identifiers

| Original GMQ item | Item wording | Motive dimension | Final GMQ-R identifier |
| --- | --- | --- | --- |
| 1 | As a way to make friends | Social | GMQ-R1 |
| 2 | To relax | Coping | GMQ-R2 |
| 3 | Because you like the feeling | Enhancement | GMQ-R3 |
| 4 | Because it’s what most of your friends do when you get together | Social | GMQ-R4 |
| 5 | To forget your worries | Coping | GMQ-R5 |
| 6 | Because it’s exciting | Enhancement | GMQ-R6 |
| 7 | To be sociable | Social | GMQ-R7 |
| 8 | Because you feel more self-confident or sure of yourself | Coping | Removed |
| 9 | To get a “high” feeling | Enhancement | GMQ-R8 |
| 10 | Because it’s something I do on special occasions | Social | GMQ-R9 |
| 11 | Because it helps when you are feeling nervous or depressed | Coping | GMQ-R10 |
| 12 | Because it’s fun | Enhancement | GMQ-R11 |
| 13 | Because it makes a social gathering more enjoyable | Social | Removed |
| 14 | To cheer up when you’re in a bad mood | Coping | GMQ-R12 |
| 15 | Because it makes you feel good | Enhancement | GMQ-R13 |

*Note.* GMQ = Gambling Motives Questionnaire; GMQ-R = Gaming Motives Questionnaire-Revised. The original GMQ item numbers refer to the initial 15-item measure. After original items 8 and 13 were removed, the 13 retained items were sequentially renumbered as GMQ-R1 to GMQ-R13.

**Appendix B. Network Accuracy and Robustness Analyses**

Bootstrap analyses were conducted to evaluate the robustness of the network estimates. For the total-sample network, nonparametric bootstrap analyses were used to assess edge-weight accuracy and edge-weight differences (Figures B1). The edge-accuracy plot showed that the bootstrapped mean estimates were generally consistent with the original sample estimates, indicating acceptable accuracy of edge-weight estimation. The edge-difference test further showed that several stronger edges could be statistically distinguished from weaker edges. These results supported the reliability of the main edge-weight pattern in the total-sample network.

**Figure B1. Edge-weight accuracy and edge-weight difference test for the total-sample network.** Panel a) presents the bootstrapped 95% confidence intervals for edge-weight estimates in the total-sample network. The red line represents the original sample estimates, black dots represent bootstrap means, and grey areas indicate confidence intervals. Panel b) presents the bootstrapped edge-weight difference test, with dark cells indicating significant differences between pairs of edge weights and lighter cells indicating non-significant differences.

To evaluate the robustness of the gender-specific motivational networks, bootstrap analyses were conducted separately for the male and female networks after adjusting for age and game type. Case-dropping bootstrap procedures were used to examine the stability of centrality indices, and nonparametric bootstrap procedures were used to assess edge-weight accuracy and edge-weight differences.

For centrality stability, the case-dropping bootstrap results indicated that the centrality estimates of both gender-specific networks were sufficiently stable (Figures B2). In the male network, the correlation stability coefficient was high for both strength, CS = 0.67, and expected influence, CS = 0.75. In the female network, expected influence also showed high stability, CS = 0.75, while strength showed acceptable stability, CS = 0.52. All CS coefficients exceeded the recommended criterion of 0.50, and the centrality indices of both the male and female networks were considered sufficiently stable.

**Figure B2. Case-dropping bootstrap results for centrality stability in the gender-specific networks.** Panel a) presents the male network, and Panel b) presents the female network. Lines indicate the average correlations between centrality estimates from subset networks and those from the original sample for strength and expected influence.

For edge-weight accuracy (Figures B3), the nonparametric bootstrap analyses showed that the bootstrapped mean edge weights were generally close to the original sample estimates in both the male and female networks, supporting the reliability of the main edge-weight patterns. For edge-weight differences (Figures B4), the edge-difference tests showed that several stronger edges were significantly different from weaker or near-zero edges in both gender-specific networks, whereas many smaller edges did not differ significantly from one another. Overall, these results indicate that the strongest edges in the male and female networks were statistically distinguishable, while differences among weaker edges should be interpreted with caution.

**Figure B3. Edge-weight accuracy in the gender-specific networks.** Panel a) presents the bootstrapped 95% confidence intervals for edge-weight estimates in the male network, and Panel b) presents the corresponding results for the female network. The red line represents the original sample estimates, black dots represent bootstrap means, and grey areas indicate 95% bootstrap confidence intervals.

**Figure B4. Edge-weight difference tests in the gender-specific networks.** Panel a) presents the bootstrapped edge-weight difference test for the male network, and Panel b) presents the corresponding results for the female network. Dark cells indicate pairs of edges that differed significantly in strength, whereas lighter cells indicate non-significant differences.
